# A Modified Vaccinia Ankara Virus (MVA) Vaccine Expressing African Horse Sickness Virus (AHSV) VP2 Protects Against AHSV Challenge in an IFNAR −/− Mouse Model

**DOI:** 10.1371/journal.pone.0016503

**Published:** 2011-01-26

**Authors:** Javier Castillo-Olivares, Eva Calvo-Pinilla, Isabel Casanova, Katarzyna Bachanek-Bankowska, Rachael Chiam, Sushila Maan, Jose Maria Nieto, Javier Ortego, Peter Paul Clement Mertens

**Affiliations:** 1 Institute for Animal Health, Pirbright, Woking, Surrey, United Kingdom; 2 Centro en Investigacion y Sanidad Animal (CISA), Valdeolmos, Madrid, Spain; 3 Wellcome Trust Sanger Institute, Wellcome Trust Genome Campus, Hinxton, Cambridge, United Kingdom; Veterinary Laboratories Agency, United Kingdom

## Abstract

African horse sickness (AHS) is a lethal viral disease of equids, which is transmitted by *Culicoides* midges that become infected after biting a viraemic host. The use of live attenuated vaccines has been vital for the control of this disease in endemic regions. However, there are safety concerns over their use in non-endemic countries. Research efforts over the last two decades have therefore focused on developing alternative vaccines based on recombinant baculovirus or live viral vectors expressing structural components of the AHS virion. However, ethical and financial considerations, relating to the use of infected horses in high biosecurity installations, have made progress very slow. We have therefore assessed the potential of an experimental mouse-model for AHSV infection for vaccine and immunology research. We initially characterised AHSV infection in this model, then tested the protective efficacy of a recombinant vaccine based on modified vaccinia Ankara expressing AHS-4 VP2 (MVA-VP2).

## Introduction

African horse sickness (AHS) is a lethal viral disease of equids, caused by an orbivirus that is closely related to bluetongue virus (BTV). The African horsesickness virus (AHSV) is transmitted by biting midges of the genus *Culicoides,* which become infected after taking a blood meal from an infected and viraemic host. [1]. The disease is endemic to Sub-Saharan Africa but sporadic outbreaks have had devastating effects in Northern Africa, Europe, Middle East and India [2], [3], [4], [5].

The AHSV genome is composed of ten linear segments of dsRNA, encoding seven structural proteins VP1 to VP7 and four non-structural proteins NS1, NS2, NS3 and NS3a [6]. The AHSV particle is organised as three concentric layers of proteins. The outer capsid, which is composed of two proteins, VP2 and VP5, interacts with neutralizing antibodies that are generated during infection of the mammalian host. There are nine distinct serotypes of AHSV, which can be distinguished in virus or serum neutralisation tests (VNT or SNT). The identity of each serotype is controlled primarily by the amino acid sequence of VP2, which contains the majority of neutralising epitopes and is the principal serotype-specific antigen of AHSV [7], [8]. Animals that survive infection by a single AHSV serotype are subsequently protected against the homologous type, although they can still be infected by the other serotypes. The AHSV core consists of two major proteins that form distinct capsid layers: VP7 forms the core surface layer; while VP3 forms the innermost ‘subcore’ shell. The subcore also contains three minor proteins, VP1, VP4 and VP6 that form core associated transcriptase complexes, and surrounds the 10 segments of the viral genome (numbered segment 1 to segment 10 [Seg-1 to Seg-10] in order of decreasing molecular weight)[6].

One of the most effective intervention strategies to combat AHS is vaccination, allowing horses to survive in endemic regions. Live attenuated strains of AHSV that were developed as vaccines have been available for more than 60 years and are still routinely used in South Africa and other endemic countries [9]. However this type of vaccines causes viraemia in the host and therefore has the potential to be transmitted in the field [10]. Recent experience with similar ‘live’ BTV vaccines in Europe shows that they can also exchange genome segments (reassort) with field strains [11], [12], potentially resulting in reversion to virulence. Since these live vaccines work by causing ‘infection’ in the host, it is also difficult, or impossible, to design serological assays that will reliably distinguish (naturally) infected and vaccinated animals (‘DIVA’), making surveillance more difficult or more expensive. These drawbacks are considered to make the live vaccines unsuitable for use in the naïve host populations in non-endemic geographic regions such as Europe [13].

For these reasons a number of recombinant vaccination strategies have been pursued over the years, as an alternative to attenuated vaccines. Baculovirus expressed AHSV capsid proteins VP2, VP5, VP7 and VP3, either individually or combined to form virus-like particles (VLP), have shown promising results [14], [15], [16], [17]. The use of recombinant pox-virus vectors expressing these AHSV proteins have also shown potential as vaccines for AHS [18], [19].

Although more work is needed to improve vaccines against this disease, progress is hampered by the need to perform vaccine efficacy studies in horses. Work with AHSV infected horses in high biosecurity installations represents a logistical and financial burden. For these reasons we have initiated a programme of research based on developing an experimental mouse model for AHS, based on recent successes with a similar model for bluetongue [20], [21].

We present the results of a pilot study using a small animal model for AHS, based on interferon-α receptor knock-out (IFNAR −/−) mice, and an exploration of the suitability of this system to test AHS vaccine efficacy. We have used a recombinant modified vaccinia Ankara virus, expressing AHS-4 VP2 (MVA-VP2), that was previously shown to induce a virus neutralising antibody response in ponies [19].

## Results

### a) Characterisation of AHSV-4 infection in IFNAR −/− mice. Virus dose range finder

An experimental infection with AHSV-4 was conducted in IFNAR −/− mice using two different virus dose levels. Thus, two groups of five mice were infected respectively with 10^5.8^ (Group A) or 10^4^ pfu (Group B) per mouse via the subcutaneous route. Two uninfected mice were kept as controls (Group C). Clinical signs were monitored and virus titres in blood samples determined.

All mice infected in group A presented clinical signs, which were evident by day 4 post-infection. These included presentation of rough hair coat, a hunched posture and reduction of mobility, which in some cases led to lethargy. Lacrimation and swelling of the eyelids was also observed. Neurological signs were also seen including ataxia, with the presentation of ‘circling gait’, and paresis of one, two or all four limbs often leading to paralysis. One animal from this group died of the disease, whilst two others had to be euthanized on humane grounds due to paralysis and ataxia. In contrast, mice in group B presented a milder clinical syndrome characterised by reduction of mobility, which was common to all animals, and eye swelling in just one individual. There were no neurological signs present and all animals infected in this group survived. Controls in group C remained healthy until the end of the study.

**Figure 1:**
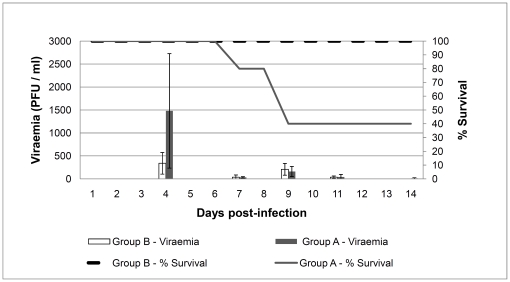
Survival rates and viraemia in IFNAR −/− mice infected with AHSV. Groups of five mice were infected with AHSV-4 using 10^4^ (Group A) or 10^5.8^ (Group B) pfu/mouse. Viraemia is represented by histograms expressing mean pfu/ml values of each of the samples collected for each day. Survival rates are represented by lines and express the percentages of surviving mice per day.

Mice in group A developed a significantly higher viraemia (mean virus titres) than those in group B (Fig. 1). Furthermore, animals with the highest viraemia titres (ranging from 794 to 2,818 pfu/ml) at the peak of the infection on day 4, either died or presented severe neurological signs, indicating a correlation between the level of viraemia and the severity of the infection. The statistical analysis of the percentage of survival between groups A and B using the Logrank test indicated significant differences (p<0.05). Pathological examinations were performed on the three mice in group A that did not survive the experiment. Macroscopic lesions were present in the spleen, liver, kidneys, brain and lungs. In each case the spleen appeared congested and very enlarged, approximately 3 times larger than normal. Kidneys and liver were also congested and enlarged. Lungs appeared congested and in some cases surface ecchymotic haemorrhages were observed. The brain of these animals looked slightly congested, almost normal.

**Figure 2:**
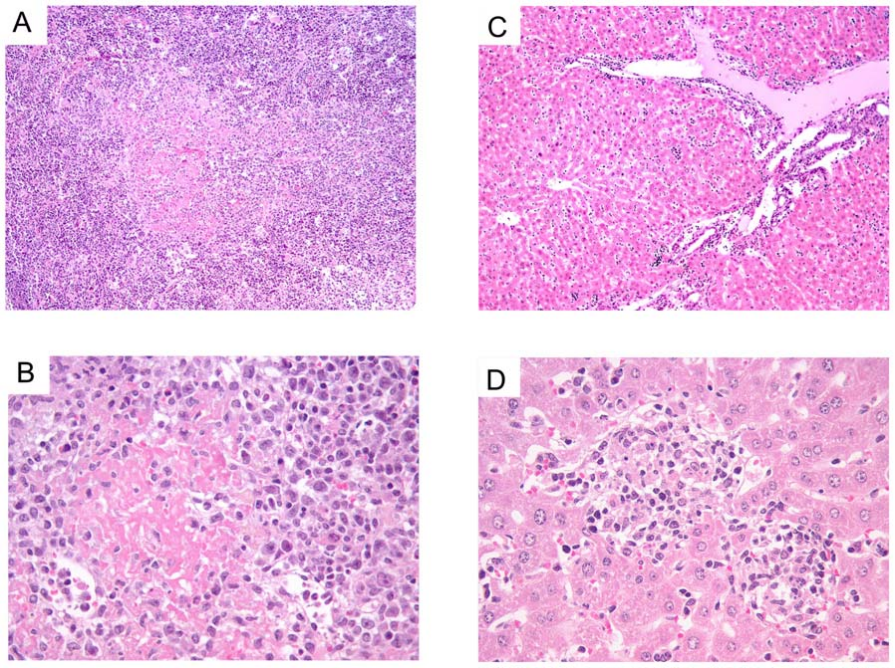
Histopathology of spleen and liver from AHSV-4 infected IFNAR −/− mice. **Liver and Spleen. Hematoxylin-eosin staining.** Representative examples of the microscopic lesions found on liver and spleen of AHSV-4 infected IFNAR −/− mice are shown. Spleen and liver samples were collected from IFNAR −/− mice, on day 7 post-infection with AHSV-4. The spleen shows loss or its normal structure (a) and abundant deposits of amyloid (a,b). The liver presents a mononuclear inflammatory infiltrate in the portal triad (c) and foci of necrosis (d).

**Figure 3:**
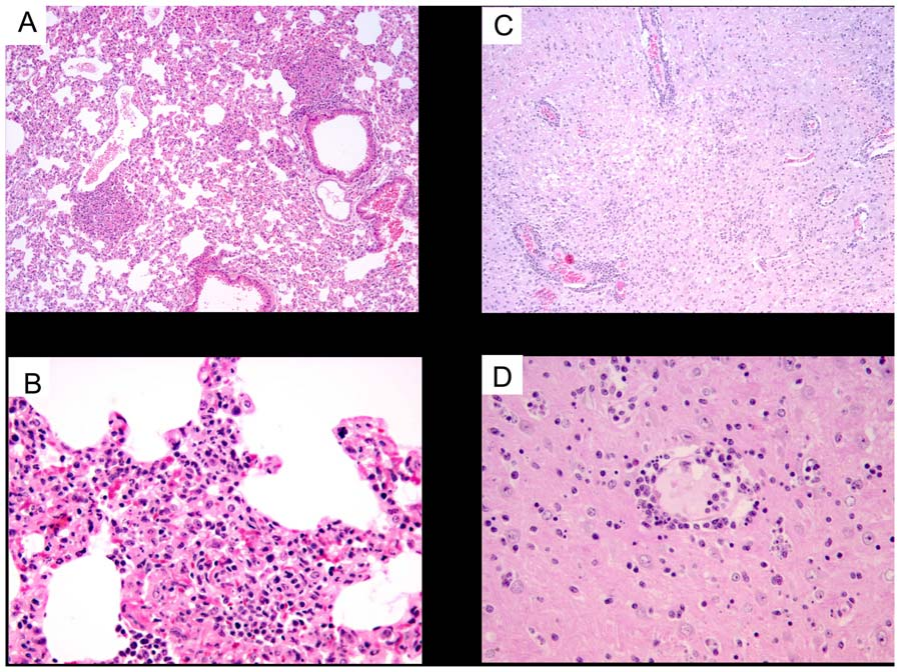
Histopathology of AHSV-4 infected IFNAR −/− mice. **Lung and Brain.** **Hematoxylin-eosin staining.** Representative examples of the microscopic lesions found on lung and brain of AHSV-4 infected IFNAR −/− mice are shown. Spleen and liver samples were collected from IFNAR −/− mice, on day 7 post-infection with AHSV-4. The lung tissue presented hyperemia, enlargement of the interalveolar septa and a pronounced mononuclear infiltrate (a,b). Images of brain show areas of microgliosis (c) and perivascular cuffing of mononuclear cells (c,d).

Microscopically, the spleen showed a loss of its normal structure and it was not possible to observe the separation between red and white pulp due to a marked lymphoid depletion (Fig. 2a, b). In addition, there was abundant presence of amyloid. Two main features were observed in the liver, namely the presence of a rich inflammatory mononuclear infiltrate in the periportal spaces as well as randomly distributed foci of necrosis. The latter consisted of cell debris together with apoptotic and necrotic hepatocytes surrounded by mononuclear cells (Fig. 2c, d). The lung tissues presented enlarged interalveolar spaces which were filled with an abundant mononuclear cell infiltrate, consistent with a viral interstitial pneumonia (Fig. 3a, b). All three mice presented meningoencephalomyelitis, which was significantly less marked in the animal that died earlier on day 7 than in the two euthanased a day later. There was a marked inflammatory mononuclear infiltrate in the meninges, presence of perivascular leukocyte cuffs randomly distributed in the brain parenchyma as well as multiple foci of microgliosis and neuronal apoptosis (Fig. 3c, d).

### b) Protective efficacy of MVA-VP2 vaccinated IFNAR −/− against AHSV-4 challenge

Once the challenge infection of IFNAR −/− mice with AHSV-4 had been characterised and that doses in the order of 10^6^ pfu/mouse induced clinical signs, viraemia and significant levels of lethality, we evaluated the protective efficacy of a recombinant MVA expressing VP2 of AHSV-4 (MVA-VP2). In earlier studies this recombinant MVA virus was shown to induce virus neutralising (VN) antibodies against AHSV-4 in ponies [19]. Six IFNAR −/− mice (M1–M6) were vaccinated by the intra-peritoneal route on days 0 and 28 with 10^7^ pfu of MVA-VP2 per mouse. These animals and six unvaccinated IFNAR −/− control mice (C1–C6) were subsequently challenged on day 35 with 10^6^ pfu of AHSV-4. Blood samples were collected at regular intervals for virus titration and assessment of antibody responses by VN assays and VP7- ELISA. The clinical signs, percentage survival, virus titres and Ct values determined by real-time RT-PCR in blood, were compared between the two groups of animals, to evaluate the level of protective immunity afforded by MVA-VP2 vaccination.

**Figure 4:**
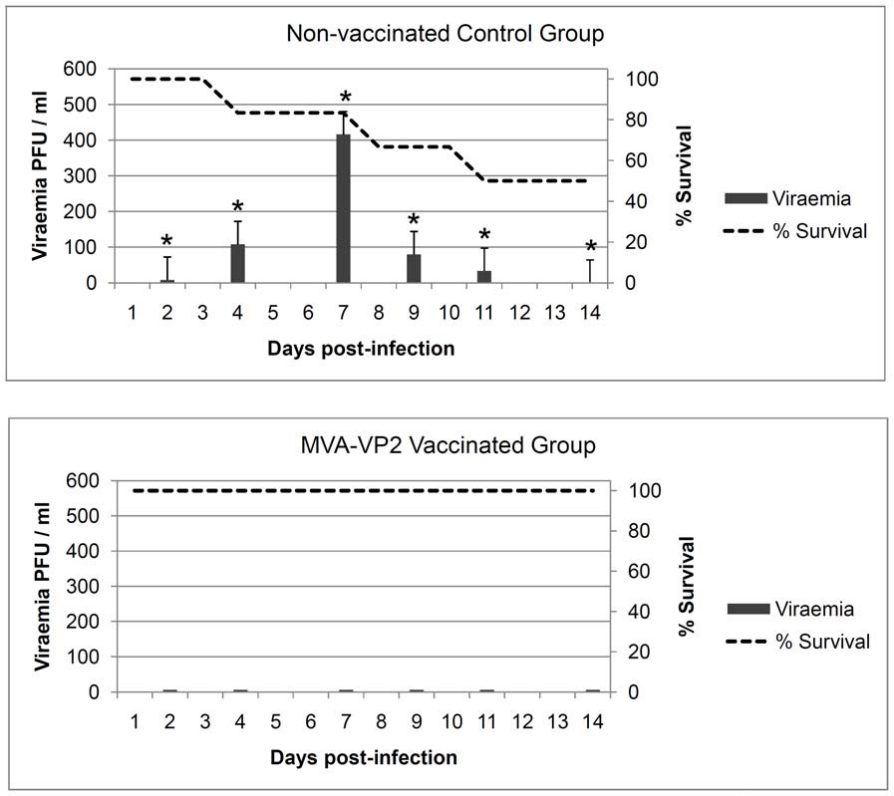
Protection of MVA-VP2 vaccinated IFNAR −/− mice against AHSV-4 challenge. Non-vaccinated control and MVA-VP2 vaccinated mice were challenged with AHSV-4. Survival rates (dotted lines) and viraemia titres (grey histograms) of both groups were compared. Each histogram represents the mean values of the viral titer of six animals, and standard deviations are shown as error bars. Asterisks indicate statistical significance calculated using the Student's t test (p<0.05).

The percentage survival and viraemia showed that all of the vaccinates survived the challenge and were negative for viraemia (Fig. 4).In contrast, the control mice group presented a survival rate of 50% and were viraemic for a number of days post-infection. The statistical analysis of the survival rates using the Logrank test indicated significant differences (p<0.05) between immunized and nonimmunized groups. All of the MVA-VP2 vaccinated animals were also protected from clinical signs (data not shown) and were completely healthy until the end of the study period. In contrast, all the unvaccinated control mice developed clinical signs similar to those described earlier during the previous challenge experiment. One of the unvaccinated control animals died on day 4 and two others were euthanized due to severe neurological signs on days 8 and 11 post-infection. The statistical analysis of the survival rates using the Logrank test indicated significant differences (p<0.05) between immunized and non-immunized groups. Likewise, comparison of viraemia titres between vaccinates and controls showed statistical significance by the Student's t-test (p<0.05).

**Figure 5:**
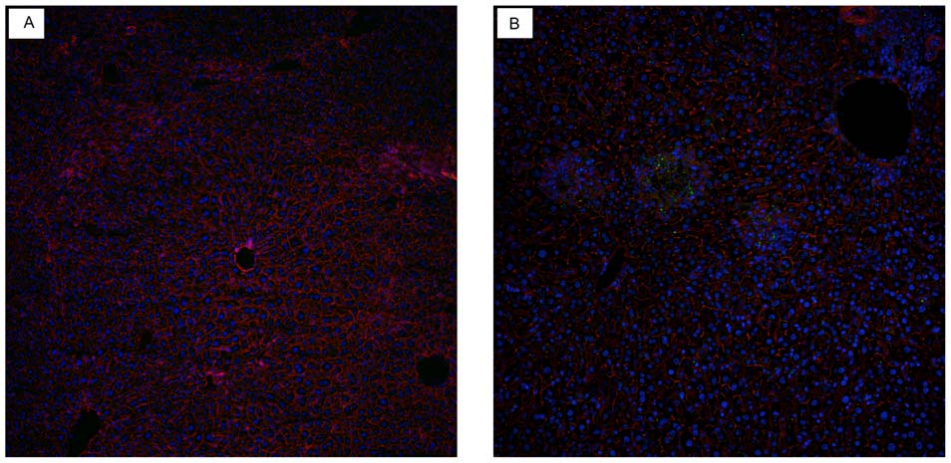
Detection of AHSV- antigen in liver tissues of AHSV-4 infected IFNAR −/− mice. Livers of normal uninfected (a) and AHSV-4 infected (b) mice were processed for immuno-fluorescence as indicated in Materials and Methods. The presence of AHSV antigen in the liver (green fluorescence) accumulates in foci of cells that resemble the foci of necrosis observed in hematoxylin-eosin sections.

**Figure 6:**
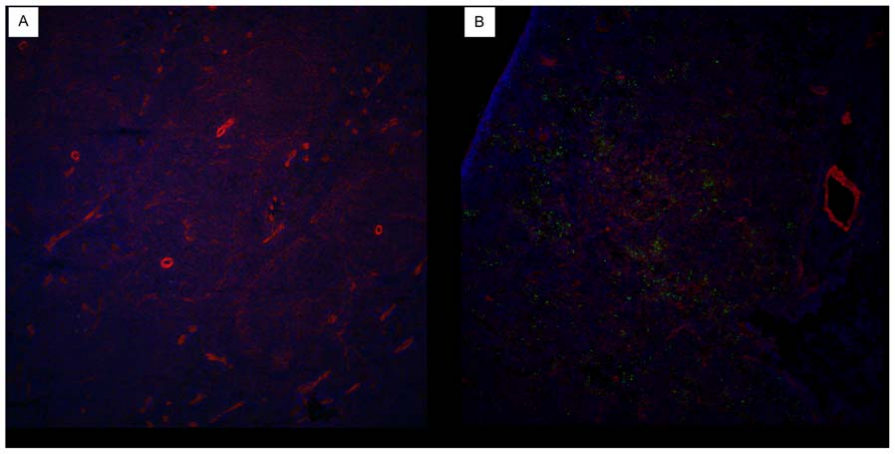
AHSV- antigen in tissues of AHSV-4 infected mice. Spleen. Spleens of normal uninfected (a) and AHSV-4 infected (b) mice were processed for immuno-fluorescence as indicated in Materials and Methods. Antigen staining (green fluorescence) can be observed ramdomly distributed throughout the spleen parenchyma.

Post-mortem examination of control mice revealed the same type of lesions as observed in the previous challenge experiment performed with AHS-4. In addition to hematoxylin-eosin staining, immuno-fluorescence experiments were used to demonstrate the presence of AHSV antigen in tissues. These were performed on paraformaldehyde fixed cryosections of AHSV-4 challenged mice and uninfected control mice. A rabbit polyclonal antiserum raised against AHSV- VP7 was used as primary antibody. Distribution of antigen in spleen was widespread and uniform, whereas in the liver it was observed as scattered foci (Fig. 5 and 6).

Data from a semi-quantitative analysis of the virus content in blood samples by real-time RT-PCR were compared with virus isolation data (Table 1). Only two of the vaccinated mice produced a Ct value for 1 and 2 days respectively. In contrast, all except one of the blood samples collected from control mice, were positive by the RT-PCR assay.

**Table 1:** Detection of AHSV-4 RNA by RT-PCR in blood samples from control (C1-C6) and MVA-VP2 vaccinated (M1-M6) animals after challenge.

Days post-infection														
Animals	1	2	3	4	5	6	7	8	9	10	11	12	13	14
C1	n.s.	37.33	n.s.	**27.56**	n.s.	n.s.	**30.29**	n.s.	**29.04**	n.s.	32.87	n.s.	n.s.	30.88
C2	n.s.	32.64	n.s.	**33.08**	n.s.	n.s.	**26.68**	n.s.	**29.17**	n.s.	**30.28**	n.s.	n.s.	0
C3	n.s.	37.25	n.s.	**31.44**	n.s.	n.s.	**28.54**	n.s.	**30.75**	n.s.	**30.53**	n.s.	n.s.	32.05
C4	n.s.	**34.86**	n.s.	**25.97**	n.s.	n.s.	† n.s.	n.s.	n.s.	n.s.	n.s.	n.s.	n.s.	n.s.
C5	n.s.	35.67	n.s.	**30.57**	n.s.	n.s.	**27.44**	n.s.	† n.s.	n.s.	n.s.	n.s.	n.s.	n.s.
C6	n.s.	30.57	n.s.	**31.67**	n.s.	n.s.	**28.71**	n.s.	28.77	n.s.	† n.s.	n.s.	n.s.	n.s.
M1	n.s.	0	n.s.	0	n.s.	n.s.	0	n.s.	0	n.s.	0	n.s.	n.s.	0
M2	n.s.	0	n.s.	35.58	n.s.	n.s.	37.45	n.s.	0	n.s.	0	n.s.	n.s.	0
M3	n.s.	0	n.s.	38.13	n.s.	n.s.	0	n.s.	0	n.s.	0	n.s.	n.s.	0
M4	n.s.	0	n.s.	0	n.s.	n.s.	0	n.s.	0	n.s.	0	n.s.	n.s.	0
M5	n.s.	0	n.s.	0	n.s.	n.s.	0	n.s.	0	n.s.	0	n.s.	n.s.	0
M6	n.s.	0	n.s.	0	n.s.	n.s.	0	n.s.	0	n.s.	0	n.s.	n.s.	0

Results are expressed as Ct values. Values of 0 indicate no Ct value was recorded.

Results in bold indicate that virus was isolated in tissue culture for that particular sample on that day.

† : Death.

n.s.: no sample collected.

**Figure 7:**
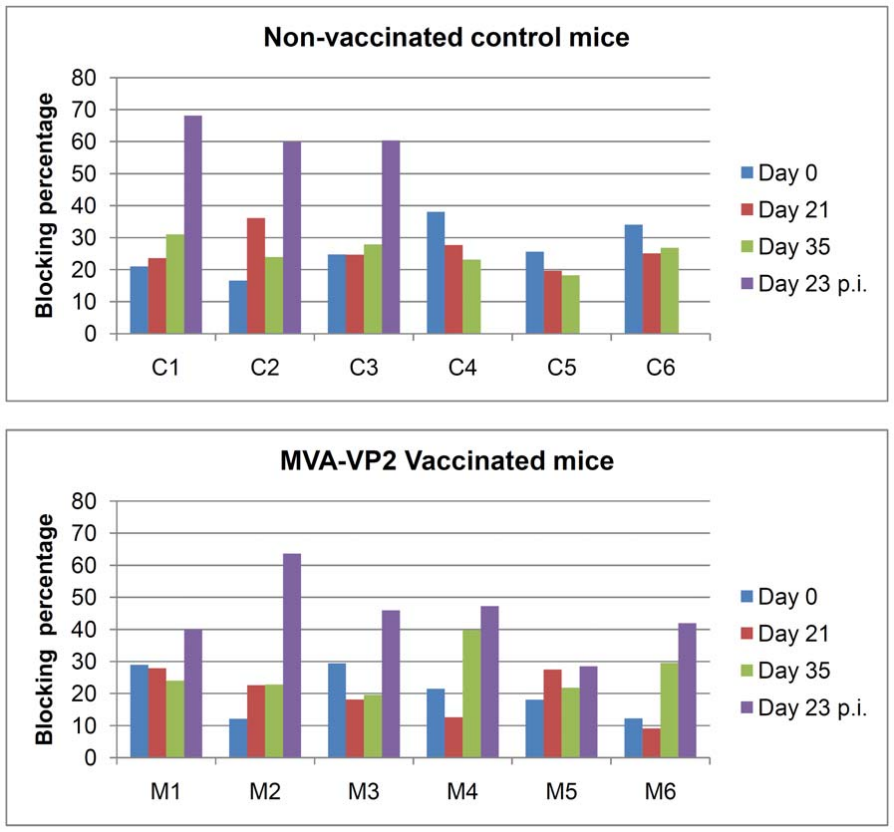
AHSV-VP7 specific antibody responses of unvaccinated and MVA-VP2 vaccinated IFNAR −/− mice after vaccination and challenge with AHSV-4. Serum samples collected from the mice at days 0 (first vaccination), 21(second vaccination), 35(challenge) and 23 post-challenge (23 p.i.) were analysed by the VP7-ELISA. Results are expressed as percentage of blocking and are represented by histogram bars. The positive threshold for a positive result is set at 50% and is represented in the graphs by a red horizontal line.

Analysis of antibody responses was performed by VN test and VP7-ELISA. All of the mice vaccinated with MVA-VP2 developed VN antibodies after the second dose of vaccine was given (Table 2). Following challenge, both the vaccinated and surviving control mice presented VN antibodies on day 23 post-infection (data not shown). The results of the VP7-ELISA assay of the serum samples from the vaccinated and challenged mice, are displayed in figure 7. As expected, all of the mice, from both groups, were negative for VP7-specific antibodies up to the day of challenge. The three surviving controls all developed VP7-specific antibodies by day 23 post-infection, indicating the animals immune system responded to the antigenic exposure. In contrast, only one of the vaccinated mice became positive by ELISA after challenge. This animal, MVA-VP2 (2), was the vaccinate whose blood was positive by RT-PCR on two consecutive days. These results indicated that all of the vaccinates, except M2, developed ‘sterile’ immunity to AHSV-4(09) reducing virus replication to such low levels that no-detectable immune response was developed to AHSV-VP7.

**Table 2:** Virus neutralisation titres of serum samples collected from the control (C1 – C6) and vaccinated (M1 – M6) mice at 4 different time points during the study.

Animals	Day 0	Day 28	Day 35	Day 23 p.i.
C1	neg	neg	neg	251
C2	neg	neg	neg	126
C3	neg	neg	neg	112
C4	neg	neg	neg	†
C5	neg	neg	neg	†
C6	neg	neg	neg	†
M1	neg	neg	5*	112
M2	neg	neg	5	447
M3	neg	neg	25	56
M4	neg	neg	14	63
M5	neg	neg	16	100
M6	neg	neg	5*	56

p.i. post-infection (neg) indicates negative result for neutralization.

(*) Indicates that samples could not be titrated due to the low volume of serum.

(†) Samples not available due to death of the animal during challenge.

## Discussion

Most of the recent African horse sickness research has focused on development of vaccine technologies that are safer, effective and compatible with their application in non-endemic countries. However, any new vaccine need to be evaluated in protective efficacy studies. Such experiments are difficult to perform in horses (the target species), due to high costs and logistic difficulties relating to the use of statistically significant number of animals in high bio-containment. For these reasons we have tested a mouse model for AHS, similar to that recently used for bluetongue [20], [21]. This system has the potential to dramatically increase research outputs and facilitate the testing of scientific hypotheses related to pathogenicity, immunology and vaccinology, which can then be verified with studies in the target species.

Laboratory animals, notably mice, have been used for many years to study infections with AHSV. Intracerebral inoculation of suckling mice is one of the primary techniques used to isolate AHSV from clinical specimens. But apart from this diagnostic application, mouse experimental mouse models have also been used in studies of AHSV pathogenicity and vaccination [22], [23]. These experiments described in these studies were previously performed in Balb/C mice although the results varied depending on the strain of virus used and lethality was often very low or absent in most cases. On some occasions, when the virus was administered by an intravenous or intranasal routes, a higher level of fatalities was observed that was sufficient for vaccine efficacy studies. However, these routes of infection are very different to that used by *Culicoides* transmitted AHSV in the natural host.

Recently, Calvo-Pinilla and co-workers successfully used IFNAR −/− mice as a model for bluetongue [20], [21]. In their studies, BTV infected IFNAR −/− mice, but not C57BL/6 mice, developed consistently clinical signs, with high mortality and high virus titre viraemia. The pathology in these animals appeared to be similar to that found in the natural host. We have explored the potential of this animal system for studies of AHSV infection and found that mortality rates, clinical signs and viraemia levels are appropriate for vaccine efficacy studies. Furthermore, the pathology of the AHSV infected mice, with the exception of the central nervous system lesions, resemble those found in AHSV infected horses. Hemorrhages and inflammatory changes in the lung, splenomegalia and congestion of other internal organs such as the liver are common pathological findings in horses infected with AHSV [1], [24]. Furthermore, immuno-fluorescence results of AHSV infected IFNAR −/− mouse tissues, demonstrated the expression of AHS antigens particularly in the spleen, suggesting this organ contains a high viral load. This feature is probably shared with AHSV infected horses, since the spleen is the preferred organ for making an etiological diagnosis of AHS in the horse (post-mortem) (OIE, 2010). The presence of neurological signs and lesions in the nervous system is a feature that has not frequently been observed in AHSV infected horses. However, the virus used for challenge in these experiments derives from a mouse brain isolate of AHSV and may have retained/aquired a certain level of neurotropism. It has to be mentioned that most isolates of AHSV are derived from mouse brain, since this has been traditionally the preferred virus isolation method. This is particularly true for the reference strains of AHSV, such as the virus we have used for our studies.

The Madrid 87 strain used in our studies was chosen because the VP2 gene employed to construct the MVA-VP2 vaccine virus was derived from it. This virus was initially isolated in mouse brain, further passaged once in mouse brain and three times in BHK before used in our mouse experiments. This virus strain, although obviously not exactly the same stock, was used in challenge experiments in horses inducing typical severe signs of AHS including death (10).

We used a subcutaneous route of infection because it most closely resembles the natural route of entry/infection during the natural AHSV infection cycle. BTV infection of IFNAR −/− mice via this route induces similar levels of lethality, viraemia and pathology to those generated via an intravenous route (Javier Ortego, personal communication). However, it is uncertain whether the route of challenge would influence the outcome of AHS virus infection in IFNAR −/− mice. Vaccination was performed by the intraperitoneal route since this is a well established route and it had been used successfully in IFNAR −/− mice vaccinated with recombinant MVA (20).

Interferon alpha (IFN-α) can play an important role in the innate immune response against viruses. IFN-α is produced by plasmacytoid dendritic cells in response to viral dsRNA, which binds to cellular receptors of neighbouring cells, stimulating of a series of antiviral mechanisms that help to clear infection. It is not known yet whether AHSV specifically induces IFN-α, or if IFN-α plays an important role in controlling AHSV infection, but evidence from studies with BTV suggest this possibility [25]. Our study also points towards this, since previous experimental infection of Balb/C mice with AHSV by the subcutaneous route consistently failed to induce clinical signs or mortality [23], which contrasts with the significant pathology and mortality (50%) observed in AHS virus infected IFNAR −/− mice. However, more work, is needed to elucidate the interaction of AHSV with the host's innate immune system.

One concern over the use of IFNAR −/− mice in vaccination studies is the potentially detrimental effect that the absence of IFN-α signalling could have on the adaptive immune response of the host. However, vaccination studies in IFNAR −/− mice with non-infectious agents, such as recombinant vesicular stomatitis virus glycoprotein, have shown that IFNAR −/− mice can develop normal levels of neutralising antibodies and cytotoxic T-lymphocyte responses [26]. Mice lacking IFN-α responses could not cope with most viral infections [27] but the lack immune responses observed in these animals, such the abscence of a cytotoxic T-lymphocyte response of lymphochoriomeningitis virus infected IFNAR −/− mice, was not due to a deficient immune system. Likely, it is a reflection of the exhaustion of virus-specific activated T cells following exposure to high doses of rapidly replicating virus, favored by the absence of IFN-α [28]. From this it follows that vaccination of IFNAR −/− mice with a highly attenuated virus will result in a complete immune response. This has been described for IFNAR −/− mice infected with the attenuated Lancy strain of vaccinia virus [26].

In our studies we have used the highly attenuated vaccinia MVA strain as a vaccine vector and showed that IFNAR −/− mice vaccinated with MVA-VP2 developed VN antibodies and protective immunity against AHSV. The levels of immunity are very high since none of vaccinates presented viraemia and only two animals produced blood samples that were positive by highly sensitive real-time RT-PCR techniques. Furthermore, only one vaccinate developed VP7 specific antibodies after challenge suggesting that the vaccinates were exposed to only very low levels of virus antigen. Our data confirms that a lack of IFN-α in mice did not affect the immune responses elicited by vaccination with an MVA based vaccine. These findings are consistent with similar studies performed with MVA based vaccines against BTV [20].

The protective effect against AHSV infection that was provided by MVA-VP2 vaccination was not surprising. We have previously demonstrated induction of VN antibodies in ponies vaccinated with the same recombinant virus [19] and others have succeeded in protecting horses against AHS using VP2 based vaccines, either in the form of baculovirus expressed protein [14], [16], [17] or pox-virus vectors [18], [29]. VP2 is the main target of neutralising antibodies on AHSV, although neutralising epitopes have also been identified on VP5 of AHS virus serotype 4 [30] and this protein seems to exert some conformational effect on VP2 epitopes. There are BTV and AHSV vaccination studies showing that co-expression of both outer capsid proteins induces a more effective protective immunity than either protein alone. Although we have not used a dual expression vector vaccine expressing VP2 and VP5, it is clear from our studies that VP2 alone was indeed able to provide full protection in the IFNAR −/− model. Moreover, vaccination and challenge studies using baculovirus expressed AHSV-5 VP2 [14], or a recombinant vaccinia virus expressing VP2 [29] showed that VP5 was not critical for induction of a protective response in horses. More work is needed to further understand the molecular nature of the VN epitopes on AHSV and the importance of VP2-VP5 interactions in induction of full protective immunity.

Our study confirms the potential MVA-VP2 as a vaccine against AHSV. This vector vaccine stimulated VN antibodies in ponies, shows protective capacity in the IFNAR −/− model and because it is based on a restricted antigenic repertoire of the virus, is suited for differential diagnostics with an appropriate differential ELISA test (DIVA strategy). A VP7-ELISA that has already been developed as a diagnostic assay for AHSV specific antibodies could serve this purpose, since the control animals that survived the infection all presented VP7 specific antibodies, in contrast to those that had only been vaccinated with MVA-VP2, which showed no response to VP7. Most of the vaccinated animals that were subsequently also challenged with AHSV, still failed to develop VP7 specific antibodies, because they did not become infected. However, in one case (M2 in Figure 7) a positive ELISA result was recorded, although this reflected a low level infection post challenge. Further studies in horses need to be performed to confirm the protective efficacy of MVA-VP2 vaccination in the target species, as well as the application of suitable differential diagnostic tests. However these experiments can be initiated knowing that the vaccinates are likely to survive and would be spared the worst clinical signs of AHS.

In conclusion, we have shown that IFNAR −/− mice can be infected by a subcutaneous route, inducing clinical signs, lethality, viraemia and pathology that are appropriate for immunology and pathogenesis studies of AHSV. To gain further evidence of the potential of this system, we conducted a vaccination and challenge experiment in this model, demonstrating the protective efficacy of the recombinant MVA-VP2 prototype vaccine. More work is needed to refine and improve this model and to determine whether our findings can be extended to other strains and serotypes of AHSV. This experimental system will facilitate work on individual and even cross-reactive vaccines as well as other aspects of AHS research.

## Materials and Methods

### Virus and cells

Baby hamster kidney cells (BHK-21), and Vero cells, obtained from the Institute for Animal Health Central Service Unit, were grown in Dulbecco's modified Eagle's medium (DMEM) supplemented with 2 mM glutamine, antibiotics and 10% fetal calf serum (FCS). Standard virus titrations were performed in Vero cells. The AHSV-4 virus stock used for challenge and for virus neutralisation tests was derived from a Spanish isolate of AHSV (Madrid-87) passaged twice in mouse brain and three times in BHK-21 cells. Virus stocks were generated by infection of confluent BHK-21 cells with this virus using a multiplicity of infection (MOI) of 0.1. When total cytopathic effect (CPE) was visible, the cells and supernatants were harvested and centrifuged. The supernatant was kept at −80C.

The MVA-VP2 virus used in this study was derived by single passage in the chicken embryo fibroblast cell line DF-1 of the construct described elsewhere [19]. The DF-1 cells were obtained from the Microbiological Services of the Institute for Animal Health.

### Mice

All experiments with live animals were performed under the guidelines of the European Community (86/609) and were approved by the Centro de Investigación en Sanidad Animal, INIA, Madrid (CISA), reference number: 2008/007. Mice were maintained under pathogen-free conditions and allowed to acclimatize to the biosafety level 3 (BSL3) animal facilities at CISA for 1 week before use in our experiments.

### Animal inoculation and processing of samples

For challenge, mice were innoculated subcutaneously with the challenge strain. Mice were examined for clinical symptoms daily. Animals showing severe symptoms were humanely euthanized. These clinical signs included: severe ataxia, paralysis, circling gait, lethargy and any other condition that affected the animals capacity to eat or drink. Whole blood was collected in EDTA from all animals at regular intervals after inoculation for virus isolation. 200 µl of blood was washed in PBS and resuspended in 900 µl of water to lyse the cells. 100 µl of 10x PBS was added to the sample and 10-fold dilutions of this material were used for quantification of viraemia using a standard plaque assay on Vero cells as described [20], [21].

### Murine immunizations

IFNAR −/− mice were immunised by two intraperitoneal injections of MVA-VP2 administered on days 0 and 28 using 10^7^ pfu/mouse. Mice were bled for serum collection on days 0, 28, 35 (day of challenge) and 23 days post-challenge.

### Serology

Sera were tested for neutralising antibodies using standard procedures using AHSV-4.Samples were preliminary screened for neutralising activity at a single dilution of 1/5. Positive samples were further titrated to determine the VN titre of the sera. Briefly, in a microtitre flat bottomed plate, 50 µl of serum samples dilutions in DMEM without bovine serum were incubated for 1 hour at 37°C with 50 µl of 100 TCID_50_ of AHSV. A virus titration was included to check the virus dose of the assay was correct and a titration of horse antisera positive for AHSV-4 VN antibodies were included in the assay as positive controls. Vero cells were added at 2×10^4^ cells/well/100 µl and plate was incubated at 37°C for 3 days. Wells were observed for development of cytopathic effect.

A commercial blocking ELISA test (Ingezim 14AHSK3, INGENASA, Madrid, Spain) was used to detect the presence of VP-7 specific antibodies in serum. Manufacturer recommendations were used.

### Histopathology

Samples from different tissues and organs were taken and fixed in 10% buffered formalin (pH 7.2) for histopathological studies. After fixation, samples were dehydrated through a graded series of alcohol to xylol and embedded in paraffin wax. Sections of 4-micron-thick were cut and stained with hematoxylin and eosin for histopathological analyses.

In addition, tissues were also cryopreserved in OCT, cut in 7 micron-thick sections, fixed in 4% paraformaldehyde and processed for immuno-fluorescence. Briefly, a rabbit antiserum raised against AHS-9 -VP7 crystals was diluted 1/1000 in PBS containing 0.5% bovine serum albumin (PBS-A) and applied to the sections. After 1 hour incubation at room temperature (RT), the slides were washed in PBS and incubated with 488-Alexafluor conjugated anti-rabbit IgG (Invitrogen) diluted 1/200 in PBS-A. A final wash in PBS preceded a 5 minute incubation step with rhodamin-phalloidin and a 5 minute incubation step with DAPI. Sections were then mounted and observed by confocal microscopy.

### RT-qPCR

Total RNA was extracted from blood samples processed as indicated above using BioRobot Universal (Qiagen). After heat denaturation the RNA was tested by real time RT-PCR on an Mx3005p machine (Stratagene, Agilent Technologies) essentially as described by Shaw *et al*. [31], using on this occassion serogroup-specific primers and probe targeting AHSV-4 genome segment 1 (assay under commercialization with LSI, France) and analysed using the manufacturer software (Stratagene, Agilent Technologies).

## References

[1] Mellor PS, Hamblin C (2004). African horse sickness.. Vet Res.

[2] Diaz Montilla R, Panos Marti P (1967). Epidemiology of AHS in Spain.. Bulletin Office International des Epizooties.

[3] Howell PG (1960). The 1960 epizootic of AHS in the Middle East and S.W. Asia.. Journal of the South African Veterinary Medical Association.

[4] Rodriguez M, Hooghuis H, Castano M (1992). African horse sickness in Spain.. Vet Microbiol.

[5] Rafyi A (1961). Horse sickness.. Bulletin Office International des Epizooties.

[6] Roy P, Mertens PP, Casal I (1994). African horse sickness virus structure.. Comp Immunol Microbiol Infect Dis.

[7] Ranz AI, Miguet JG, Anaya C, Venteo A, Cortes E (1992). Diagnostic methods for African horsesickness virus using monoclonal antibodies to structural and non-structural proteins.. Vet Microbiol.

[8] Burrage TG, Trevejo R, Stone-Marschat M, Laegreid WW (1993). Neutralizing epitopes of African horsesickness virus serotype 4 are located on VP2.. Virology.

[9] von Teichman BF, Smit TK (2008). Evaluation of the pathogenicity of African Horsesickness (AHS) isolates in vaccinated animals.. Vaccine.

[10] House C, House JA, Mebus AC, Williams JC, KKM, Gibbs EPJ (1992). A review of African horse sickness with emphasis on selected vaccines.. Annals of the New York Academy of Sciences: Tropical veterinary medicine - current issues perspectives: 1st Biennial Conference, American Society of Tropical Veterinary Medicine, San Juan, Puerto Rico, February 5-8 1991.

[11] Maan S, Maan NS, van Rijn PA, van Gennip RG, Sanders A (2010). Full genome characterisation of bluetongue virus serotype 6 from the Netherlands 2008 and comparison to other field and vaccine strains.. PLoS One.

[12] Batten CA, Maan S, Shaw AE, Maan NS, Mertens PP (2008). A European field strain of bluetongue virus derived from two parental vaccine strains by genome segment reassortment.. Virus Res.

[13] Van Dijk AA., Wernery U, Wade J, Mumford J, Kaaden O African Horse Sickness Vaccine Development..

[14] Scanlen M, Paweska JT, Verschoor JA, van Dijk AA (2002). The protective efficacy of a recombinant VP2-based African horsesickness subunit vaccine candidate is determined by adjuvant.. Vaccine.

[15] Roy P, Sutton G (1998). New generation of African horse sickness virus vaccines based on structural and molecular studies of the virus particles.. Arch Virol Suppl.

[16] Roy P, Bishop DH, Howard S, Aitchison H, Erasmus B (1996). Recombinant baculovirus-synthesized African horsesickness virus (AHSV) outer-capsid protein VP2 provides protection against virulent AHSV challenge.. J Gen Virol.

[17] Martinez-Torrecuadrada JL, Diaz-Laviada M, Roy P, Sanchez C, Vela C (1996). Full protection against African horsesickness (AHS) in horses induced by baculovirus-derived AHS virus serotype 4 VP2, VP5 and VP7.. J Gen Virol.

[18] Guthrie AJ, Quan M, Lourens CW, Audonnet JC, Minke JM (2009). Protective immunization of horses with a recombinant canarypox virus vectored vaccine co-expressing genes encoding the outer capsid proteins of African horse sickness virus.. Vaccine.

[19] Chiam R, Sharp E, Maan S, Rao S, Mertens P (2009). Induction of antibody responses to African horse sickness virus (AHSV) in ponies after vaccination with recombinant modified vaccinia Ankara (MVA).. PLoS One.

[20] Calvo-Pinilla E, Rodriguez-Calvo T, Sevilla N, Ortego J (2009). Heterologous prime boost vaccination with DNA and recombinant modified vaccinia virus Ankara protects IFNAR(-/-) mice against lethal bluetongue infection.. Vaccine.

[21] Calvo-Pinilla E, Rodriguez-Calvo T, Anguita J, Sevilla N, Ortego J (2009). Establishment of a bluetongue virus infection model in mice that are deficient in the alpha/beta interferon receptor.. PLoS One.

[22] Wade-Evans AM, Pullen L, Hamblin C, O'Hara R, Burroughs JN (1997). African horsesickness virus VP7 sub-unit vaccine protects mice against a lethal, heterologous serotype challenge.. J Gen Virol.

[23] O'Hara RS, Meyer AJ, Burroughs JN, Pullen L, Martin LA (1998). Development of a mouse model system, coding assignments and identification of the genome segments controlling virulence of African horse sickness virus serotypes 3 and 8.. Arch Virol Suppl.

[24] Coetzer JAW, Guthrie AJ, Coetzer JAW, Tustin RC (2004). African horse sickness.. Infectious Diseases of Livestock. 2nd ed.

[25] Jameson P, Schoenherr CK, Grossberg SE (1978). Bluetongue virus, an exceptionally potent interferon inducer in mice.. Infect Immun.

[26] Muller U, Steinhoff U, Reis LF, Hemmi S, Pavlovic J (1994). Functional role of type I and type II interferons in antiviral defense.. Science.

[27] van den Broek MF, Muller U, Huang S, Zinkernagel RM, Aguet M (1995). Immune defence in mice lacking type I and/or type II interferon receptors.. Immunol Rev.

[28] Zinkernagel RM, Moskophidis D, Kundig T, Oehen S, Pircher H (1993). Effector T-cell induction and T-cell memory versus peripheral deletion of T cells.. Immunol Rev.

[29] Stone-Marschat MA, Moss SR, Burrage TG, Barber ML, Roy P (1996). Immunization with VP2 is sufficient for protection against lethal challenge with African horsesickness virus Type 4.. Virology.

[30] Martinez-Torrecuadrada JL, Langeveld JP, Venteo A, Sanz A, Dalsgaard K (1999). Antigenic profile of African horse sickness virus serotype 4 VP5 and identification of a neutralizing epitope shared with bluetongue virus and epizootic hemorrhagic disease virus.. Virology.

[31] Shaw AE, Monaghan P, Alpar HO, Anthony S, Darpel KE (2007). Development and initial evaluation of a real-time RT-PCR assay to detect bluetongue virus genome segment 1.. J Virol Methods.

